# A Photoconvertible Reporter System for Bacterial Metabolic Activity Reveals That Staphylococcus aureus Enters a Dormant-Like State to Persist within Macrophages

**DOI:** 10.1128/mbio.02316-22

**Published:** 2022-09-14

**Authors:** Julia C. Lang, Elena A. Seiß, Adriana Moldovan, Mathias Müsken, Till Sauerwein, Martin Fraunholz, Andreas J. Müller, Oliver Goldmann, Eva Medina

**Affiliations:** a Infection Immunology Research Group, Helmholtz Centre for Infection Research, Braunschweig, Germany; b Institute of Molecular and Clinical Immunology, Health Campus Immunology Infectiology and Inflammation, Otto-von-Guericke-University, Magdeburg, Germany; c Department of Microbiology, Julius-Maximilians-Universität Würzburg, Würzburg, Germany; d Central Facility for Microscopy, Helmholtz Centre for Infection Research, Braunschweig, Germany; e ZB MED - Information Center of Life Science, Cologne, Germany; f Intravital Microscopy of Infection and Immunity, Helmholtz Centre for Infection Research, Braunschweig, Germany; Institut Pasteur

**Keywords:** *Staphylococcus aureus*, dual RNA-seq, intracellular survival, macrophages

## Abstract

Staphylococcus aureus is a leading cause of difficult-to-treat infections. The capacity of S. aureus to survive and persist within phagocytic cells is an important factor contributing to therapy failures and infection recurrence. Therefore, interfering with S. aureus intracellular persistence is key to treatment success. In this study, we used a S. aureus strain carrying the reporter mKikumeGR that enables the monitoring of the metabolic status of intracellular bacteria to achieve a better understanding of the molecular mechanisms facilitating S. aureus survival and persistence within macrophages. We found that shortly after bacteria internalization, a large fraction of macrophages harbored mainly S. aureus with high metabolic activity. This population decreased gradually over time with the concomitant increase of a macrophage subpopulation harboring S. aureus with low metabolic activity, which prevailed at later times. A dual RNA-seq analysis performed in each macrophage subpopulation showed that the host transcriptional response was similar between both subpopulations. However, intracellular S. aureus exhibited disparate gene expression profiles depending on its metabolic state. Whereas S. aureus with high metabolic activity exhibited a greater expression of genes involved in protein synthesis and proliferation, bacteria with low metabolic activity displayed a higher expression of oxidative stress response-related genes, silenced genes involved in energy-consuming processes, and exhibited a dormant-like state. Consequently, we propose that reducing metabolic activity and entering into a dormant-like state constitute a survival strategy used by S. aureus to overcome the adverse environment encountered within macrophages and to persist in the intracellular niche.

## INTRODUCTION

Staphylococcus aureus is one of the most successful human pathogens and is responsible for high numbers of hospital-acquired and community-acquired infections (1). This bacterium can grow and replicate freely in host tissue, and it can also invade a variety of host cells, including macrophages and survive intracellularly (2). Macrophages are usually the first line of host defense against S. aureus, as they are present in almost all tissues and are recruited in large numbers to the site of an infection. However, accumulating evidence suggests that a subpopulation of phagocytosed S. aureus is capable of circumventing the antimicrobial mechanisms and surviving intracellularly within macrophages (3–6). In the intracellular niche, S. aureus is protected from host immune defenses and antibiotics and may provide a long-term bacterial reservoir for chronic and relapsing infections (7, 8). Therefore, the elimination of intracellular S. aureus during infection is key for treatment success.

The molecular mechanisms underlying intracellular bacterial survival and persistence are far from being fully understood, and their identification can be complicated by the potential cell-to-cell variability of either the host cell or the intracellular pathogen, which can be missed in studies performed on bulk cell populations. For example, it is known that, despite the capacity of S. aureus to avoid intracellular killing within macrophages, only a proportion of all phagocytosed S. aureus is able to survive in the intracellular niche (9). Whether this is due to heterogeneity within the bacterial population in regard to the expression of virulence factors and metabolic activity or to heterogeneity within the macrophage population, with some cells being more permissive than others for intracellular bacterial survival, remains unknown. In this study, we investigated the host and pathogen mechanisms that influence the fate of S. aureus within macrophages by using a S. aureus strain carrying the mKikumeGR reporter system (S. aureus pKikume) (10). This reporter system consists of a monomeric photoswitchable fluorescent protein that emits green fluorescence in its original state and can be photoswitched to emit red fluorescence by UV or violet light (11). The photoconversion is performed by a 405 nm light pulse to switch green mKikumeGR to red mKikumeGR, and it is measured with 475 nm and 555 nm excitation, respectively. Crucial here are that the photoconversion of the fluorescence signal is irreversible and that the green fluorescent protein can only be recovered by the pathogen after *de novo* protein production (12). With this feature, it is possible to correlate the metabolic activity of intracellular bacteria with their capacity to recover the green fluorescence after photoconversion. We use S. aureus pKikume to infect macrophages and separate the subpopulations of macrophages harboring bacteria with high metabolic activity from the subpopulations harboring bacteria with low metabolic activity by cell sorting. A dual RNA-seq analysis performed to determine gene expression changes simultaneously in the host cells and the internalized bacteria in both macrophage subpopulations indicated that the fate of S. aureus within macrophages was not driven by heterogeneity within the host cells but rather by variability within the bacterial population in their capacity to adjust their transcriptional responses to intracellular microenvironments.

## RESULTS AND DISCUSSION

### Discrimination between macrophage subpopulations harboring S. aureus with either high or low metabolic activity using mKikumeGR.

**FIG 1:**
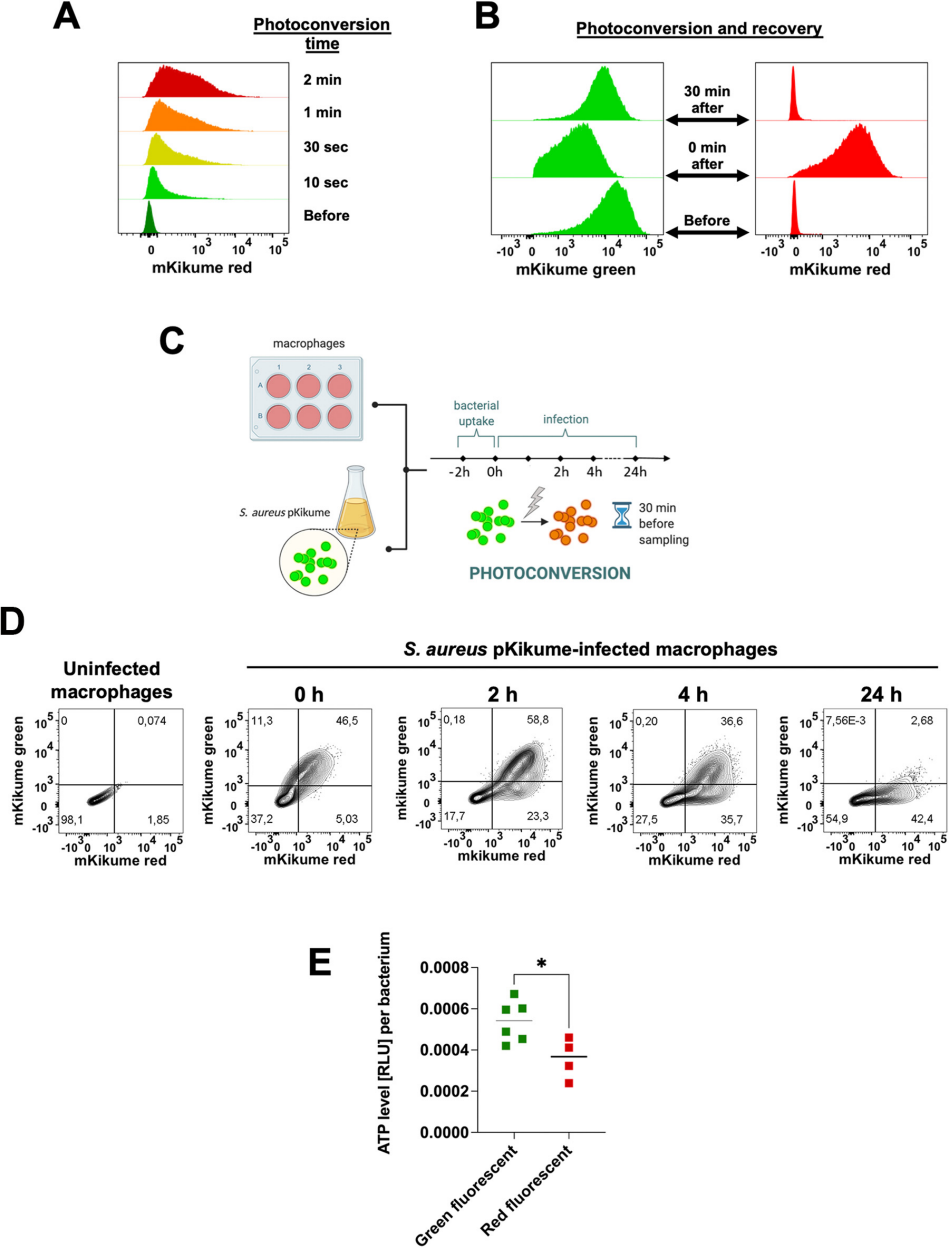
Monitoring the metabolic activity of S. aureus pKikume within macrophages. (A) Flow cytometry histograms of S. aureus pKikume, showing the photoconversion from green to red fluorescence using a 405 nm light pulse for 10 sec, 30 sec, 1 min, or 2 min. (B) Recovery of green fluorescence by S. aureus pKikume 30 min after photoconversion with a 405 nm light pulse for 90 sec. (C) Schematic representation of the experimental model in which macrophages infected with S. aureus pKikume were photoconverted and sampled at defined time intervals. Macrophages were infected with S. aureus pKikume for 2 h (bacterial uptake), treated with lysostaphin/gentamicin for 15 min to lyse the remaining noninternalized extracellular bacteria, and further incubated for 2 h, 4 h, and 24 h. Infected macrophages were subjected to a 90 sec light pulse of 405 nm wavelength to switch the bacterial fluorescence from green to red 30 min before sampling, and this was followed by a flow cytometry analysis. (D) Representative flow cytometry contour plots showing S. aureus pKikume-infected macrophages at progressing times after infection. (E) ATP levels in green or red S. aureus isolated from infected macrophages and determined by luciferase assay at 4 h postinfection. Wells with medium alone were used for background determination. ATP levels are expressed in relative light units (RLU) per viable bacterium. Each symbol represents one individual measurement from three independent experiments (*t* test, *, *P* < 0.05).

The previously described mKikumeGR-expressing S. aureus reporter system (S. aureus pKikume) (10) was used to monitor the metabolic activity of S. aureus within infected macrophages. S. aureus pKikume carries the photoconvertible fluorescence protein mKikumeGR, which emits a green fluorescence in its original state and can be switched to emit a red fluorescence by using a UV light pulse (Fig. 1A) (11). As this photoconversion is irreversible, the recovering of green fluorescence by the bacterium is only possible through the *de novo* production of fluorescent mKikumeGR protein and can therefore be used as an indicator of the bacterial metabolic activity. S. aureus pKikume can be photoconverted to red fluorescence by a 405 nm light pulse for 90 sec without affecting bacterial viability (10) and can recover the green fluorescence 30 min after the photoconversion (Fig. 1B). Intracellular S. aureus pKikume can also be efficiently photoconverted within macrophages (Fig. S1A) without affecting macrophage viability (Fig. S1B) or phagocytic capacity (Fig. S1C). Therefore, this system enables the discrimination between macrophage subpopulations harboring S. aureus either with high or with low metabolic activity by flow cytometry according to the emitted green or red fluorescence, respectively.

10.1128/mbio.02316-22.2 FIG S1 Widefield time lapse imaging of S. aureus pKikume within macrophages after photoconversion and the viability and functionality of UV-treated macrophages. Download FIG S1, PDF file, 0.5 MB. Copyright © 2022 Lang et al. 2022 Lang et al. https://creativecommons.org/licenses/by/4.0/ This content is distributed under the terms of the Creative Commons Attribution 4.0 International license.

To monitor the time-dependent metabolic activity of intracellular S. aureus, macrophages were infected with S. aureus pKikume, and the distribution of red and green fluorescence within the macrophage population was determined at progressing times postinfection by flow cytometry. Intracellular bacteria were photoconverted 30 min prior to each sampling time point. The experimental design is depicted in Fig. 1C. The results of the flow cytometry analysis indicated that directly after bacterial uptake (0 h), more than 90% of the macrophages harboring intracellular S. aureus contained bacteria with high metabolic activity, as shown by the efficient recovery of green fluorescence after photoconversion (Fig. 1D). The percentage of macrophages harboring S. aureus with high metabolic activity (green-fluorescent) decreased over time while the subpopulation of macrophages harboring S. aureus with low metabolic activity (red-fluorescent) gradually increased over the course of infection (Fig. 1D). At 24 h postinfection, more than 90% of the infected macrophages harbored S. aureus with low metabolic activity and exhibited a predominantly red fluorescence signal after photoconversion and recovery (Fig. 1D). The different metabolic activity between green-fluorescent and red-fluorescent intracellular S. aureus was corroborated by the significantly greater levels of ATP detected in the green-fluorescent bacteria versus the red-fluorescent bacteria (Fig. 1E). Hereafter in the text, we will refer to S. aureus pKikume with high metabolic activity as “green S. aureus” and to S. aureus with low metabolic activity as “red S. aureus”.

**FIG 2:**
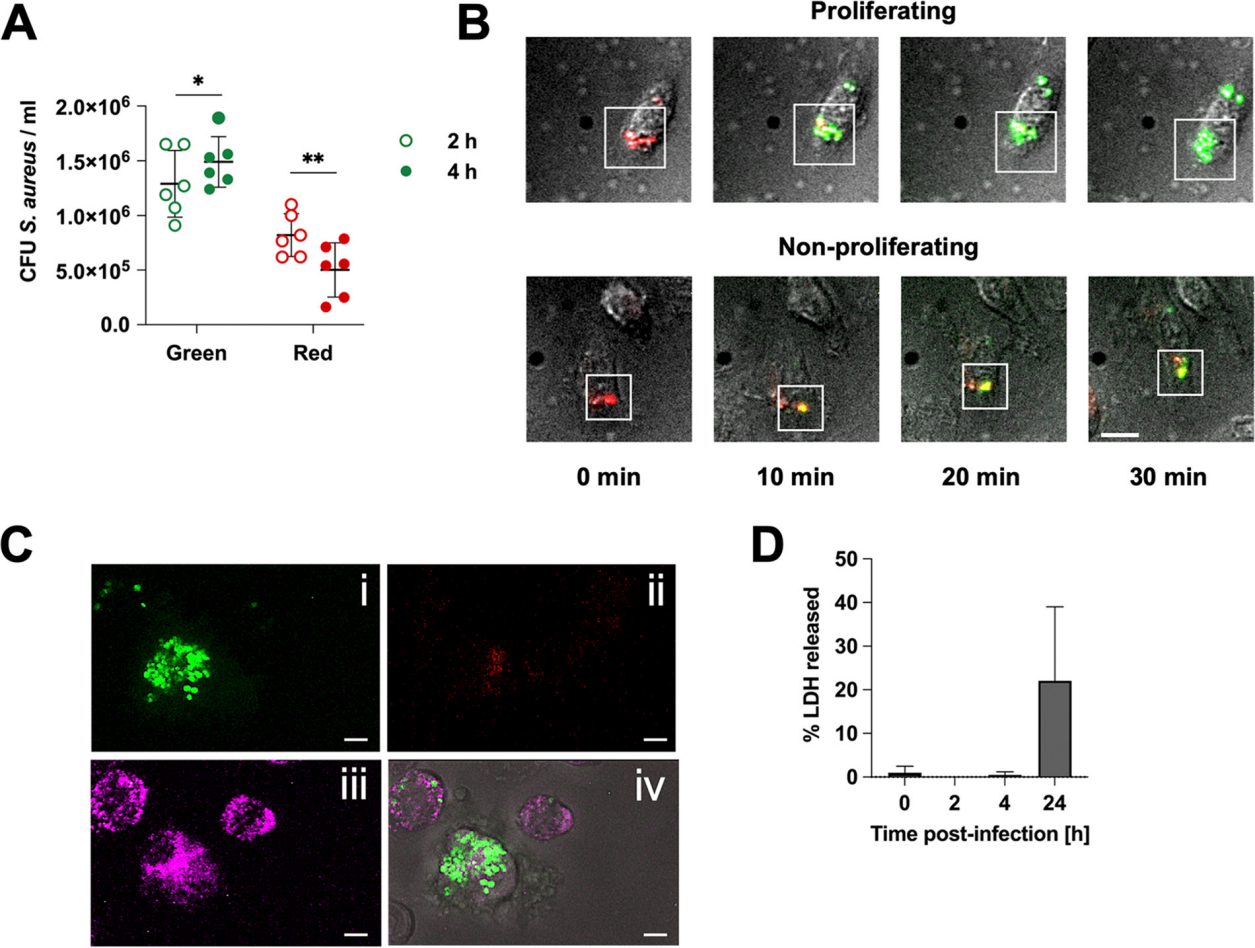
Viability and proliferation of intracellular green or red S. aureus. (A) Numbers of viable intracellular green (green symbols) or red (red symbols) S. aureus at 2 h (open symbols) and 4 h (solid symbols) postinfection. Each symbol represents an independent determination with mean values indicated by horizontal bars (*t* test, *, *P* < 0.05; **, *P* < 0.01). (B) Time-lapse imaging of photoconverted S. aureus pKikume within macrophages. The upper panels show the recovery of green fluorescence by proliferating bacteria, and the lower panels show the recovery of green fluorescence by nonproliferating S. aureus. Scale bar, 10 μm. (C) Confocal microscopy photograph of macrophages infected with S. aureus at 4 h postinfection. Bacteria exhibiting green fluorescence are shown in (i), bacteria exhibiting red fluorescence are shown in (ii), lysosomal compartments labeled using Alexa 647 conjugated dextran beads are shown in (iii), and a merged image is shown in (iv). Scale bars, 2 μm. (D) Lactate dehydrogenase (LDH) released in the culture supernatant of S. aureus*-*infected macrophages during the course of infection. Results are displayed as the percentage of the maximum LDH release achieved after the disruption of the macrophages with 0.1% Triton X-100. Each bar represents the average ± standard deviation of three independent experiments.

Next, we investigated whether the metabolic activity of intracellular S. aureus correlated with bacterial viability. For this purpose, macrophages harboring green S. aureus were separated from those harboring red S. aureus at 2 h and 4 h postinfection by cell sorting, and the viability of the intracellular bacteria was determined by plating cell lysates. The numbers of viable intracellular green bacteria slightly increased between 2 h and 4 h postinfection, indicating that only a proportion of green bacteria may undergo proliferation (Fig. 2A). Indeed, time lapse imaging microscopy of S. aureus within macrophages after photoconversion showed that green fluorescence could be recovered not only by proliferating bacteria (Fig. 2B, upper panels; Video S1) but also by bacteria which were not found to be dividing, suggesting a high protein turnover and metabolic activity in the absence of proliferation (Fig. 2B, lower panels; Video S1). The numbers of viable intracellular red S. aureus decreased between 2 h and 4 h postinfection, indicating that a proportion of bacteria were killed in this subpopulation (Fig. 2A). We also observed that approximately 2–3% of macrophages harbored high numbers of green S. aureus (Fig. 2C). Since macrophages harboring green bacteria were completely absent in the cell cultures at 24 h postinfection (Fig. 1D), we speculated that intracellular highly proliferating S. aureus could induce macrophage cell death in this time interval, as previously reported (3). Therefore, we measured the levels of lactate dehydrogenase (LDH) released in the culture supernatant of the infected macrophages as an indicator of cell death. As shown in Fig. 2D, an increased release of LDH was observed between 4 h and 24 h postinfection, indicating that a proportion of macrophages underwent cell death.

10.1128/mbio.02316-22.2 VIDEO S1 Live imaging of *S. aureus* within macrophages after photoconversion. Download VIDEO S1, MOV file, 0.3 MB. Copyright © 2022 Lang et al. 2022 Lang et al. https://creativecommons.org/licenses/by/4.0/ This content is distributed under the terms of the Creative Commons Attribution 4.0 International license.

Because the bacterial population surviving within macrophages at 24 h postinfection and beyond was in a nonproliferating status and exhibited low metabolic activity, we propose that S. aureus persists within macrophages by entering a nonreplicative dormant-like state.

### Dual RNA-seq analysis of macrophage subpopulations harboring either green or red S. aureus.

**FIG 3:**
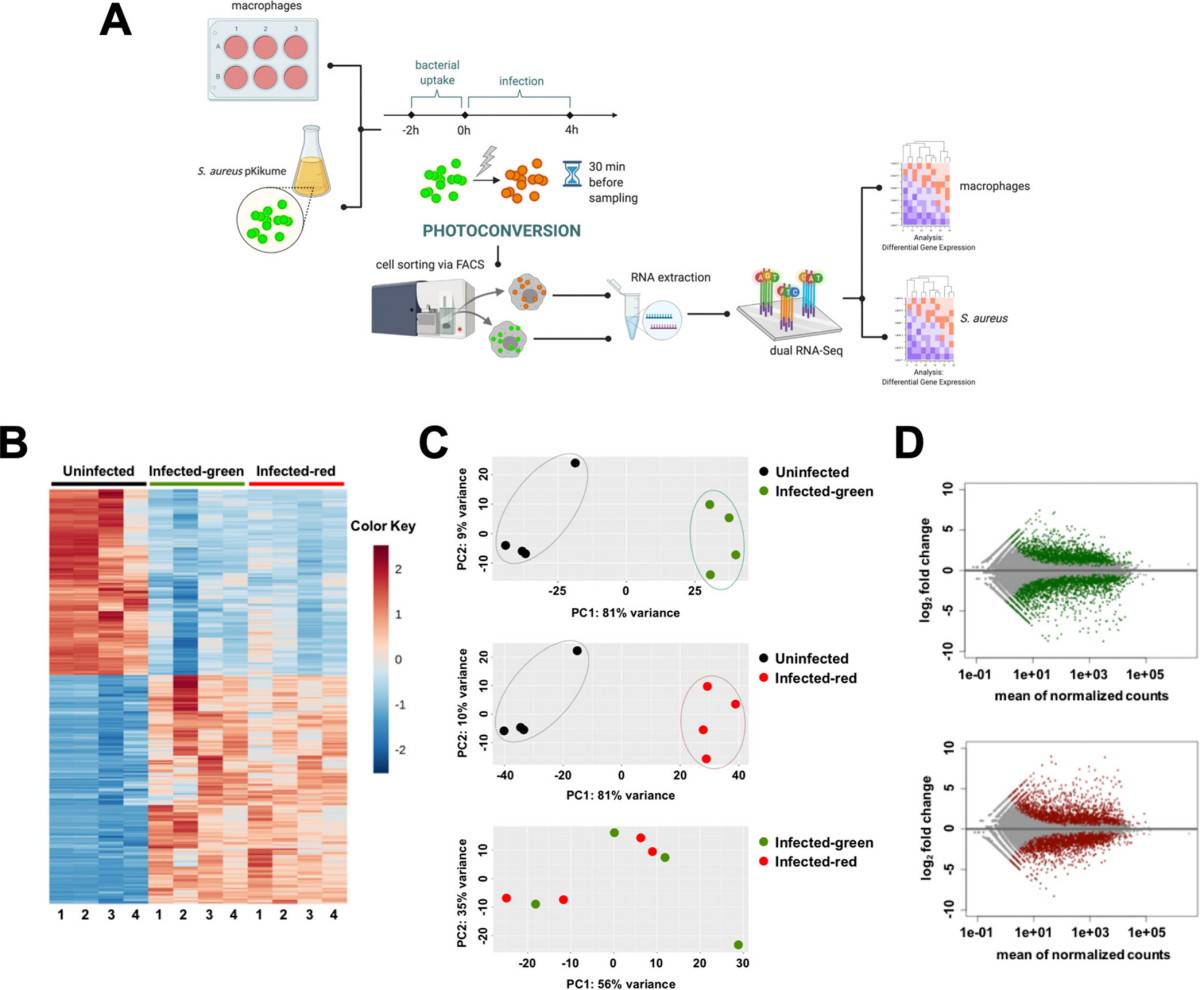
Transcriptional analysis of macrophages harboring green or red S. aureus. (A) Schematic outline of the experimental design for a dual RNA-seq analysis of the S. aureus*-*infected macrophages. The macrophages were infected with S. aureus for 2 h (bacterial uptake), treated with lysostaphin/gentamicin to lyse the remaining noninternalized extracellular bacteria (0 h), and further incubated for 4 h. Infected macrophages were subjected to a 90 sec light pulse of 405 nm wavelength to switch the bacterial fluorescence from green to red 30 min before sampling. Infected macrophages were harvested, and the subpopulations harboring green S. aureus were separated from the subpopulations harboring red S. aureus by cell sorting. RNA was isolated from both macrophage subpopulations and analyzed by dual RNA-seq to investigate the gene expression profiles of the host and the pathogen simultaneously. (B) Heat map showing gene expression levels (top 500 genes) in infected macrophage subpopulations harboring either green or red S. aureus as well as in uninfected macrophages. The color key represents the *z* score normalized transcripts per million reads (TPM). (C) PCA clustering of the transcriptomes of infected macrophages harboring green S. aureus versus uninfected macrophages (upper panel), macrophages harboring red S. aureus versus uninfected macrophages (middle panel), and macrophages harboring green versus macrophages harboring red S. aureus (lower panel) based on a Euclidean distance matrix of normalized RNA-seq data. Circles enclose replicates that cluster together. Each dot represents one biological replicate. (D) MA plots showing the log_2_-fold change in gene expression for macrophages harboring green S. aureus versus uninfected macrophages (upper panel) and macrophages harboring red S. aureus versus uninfected macrophages (lower panel). Genes with adjusted *P* < 0.05 and log_2_-fold change > 2 or log_2_-fold change < −2 are labeled in green (upper panel) and red (lower panel).

Next, we investigated whether the metabolic activity of intracellular S. aureus was driven by either heterogeneity within the macrophage population in its capacity to control intracellular bacteria or by variability within the bacterial population in its capacity to adapt to the intracellular compartments. To this end, macrophages were harvested at 4 h postinfection, the subpopulation harboring green S. aureus was separated from the subpopulation harboring red S. aureus by cell sorting, and each subpopulation was subjected to dual RNA-seq analysis. A schematic overview of the experimental setup is shown in Fig. 3A. The sampling time (4 h) was selected because approximately 50% of the infected macrophages harbored mainly green S. aureus and 50% harbored mainly red S. aureus (Fig. 1D) at this point in time. Total RNA was isolated from each macrophage subpopulation, and cDNA libraries were prepared and sequenced using an Illumina NovaSeq 6000 system. Between 144.24 and 101.46 million reads were obtained per sample, from which 25.22 to 58.33 million reads were uniquely mapped to protein-coding sequences within the Mus musculus GRCm38.p6 (GCA_000001635.26) reference genome, whereas 103.71 thousand to 19.38 million reads were uniquely aligned to the S. aureus NCTC 8325 genome (GCF_000013425.1) (Table 1).

**TABLE 1:** Number of reads mapped to the S. aureus genome and to the Mus musculus genome in uninfected macrophages (Uninfected), macrophages harboring either green (Infected-green) or red (Infected-red) S. aureus, or in S. aureus in the infection inoculum (Inoculum)

Samples	Replicate	Total no. of reads	Reads mapped to *M. musculus*	Reads mapped to S. aureus
Uninfected	1	133,226,288	58,326,032	-
Uninfected	2	107,003,438	44,306,130	-
Uninfected	3	137,140,591	46,468,501	-
Uninfected	4	117,513,868	32,530,137	-
Infected-green	1	129,078,105	44,203,733	1,593,405
Infected-green	2	129,816,704	49,424,721	2,782,439
Infected-green	3	121,394,148	25,216,555	229,794
Infected-green	4	144,239,339	40,461,111	522,922
Infected-red	1	130,349,989	35,446,167	294,425
Infected-red	2	101,461,707	37,270,062	403,429
Infected-red	3	140,321,397	42,046,848	103,711
Infected-red	4	125,287,483	39,911,330	365,333
Inoculum	1	40,035,465	-^*a*^	12,193,921
Inoculum	2	48,794,896	-	19,378,965
Inoculum	3	42,166,079	-	13,602,861
Inoculum	4	47,006,744	-	13,984,955

a Dashes indicate that none reads were mapped to the specific genome.

### Transcriptional response of infected macrophages harboring either green or red S. aureus or uninfected macrophages.

A heat map of gene expression levels in macrophages across the different samples indicated similar gene expression patterns in infected macrophages in respect to uninfected control cells, independently of the metabolic activity of the bacteria that they harbored, (Fig. 3B). The similarity in gene expression was further confirmed via a principal components analysis (PCA) of the RNA-seq data, which showed a clear separation between infected macrophages harboring either green (Fig. 3C, upper panel) or red (Fig. 3C, middle panel) S. aureus and uninfected macrophages along the first principal component (PC1). PCA clustering comparing the gene expression data sets of infected macrophages harboring green S. aureus with those harboring red S. aureus showed no clear separation between the groups, indicating a similar gene expression signature in both macrophage subpopulations (Fig. 3C, lower panel).

DESeq2 was then used to identify differentially expressed genes (DEGs) between the infected macrophage subpopulations and the uninfected controls. Genes were considered to be upregulated at a log_2_-fold change of >2 with an adjusted *P* value of <0.05, and genes were considered to be downregulated at a log_2_-fold change of <−2 with an adjusted *P* value of <0.05. A total of 522 genes were found to be upregulated, and 499 genes were found to be downregulated in macrophages containing green S. aureus, relative to uninfected control macrophages (Fig. 3D, upper panel and Table S1). In macrophages containing red S. aureus, 429 genes were found to be upregulated, and 405 genes were found to be downregulated, relative to uninfected controls (Fig. 3D, lower panel and Table S2). A KEGG pathway enrichment analysis was performed to obtain more information on the functional organization of the DEGs in both macrophage subpopulations. An analysis of the upregulated genes showed that biological processes associated with the inflammatory response, such as cytokine-cytokine receptor interactions, the TNF signaling pathway, the toll-like receptor signaling pathway, chemokine signaling, and pathways associated with infections such as influenza A, malaria, and tuberculosis were found to be enriched in both macrophage subpopulations (Table 2). No DEGs were found when comparing infected macrophages harboring green S. aureus with macrophages harboring red S. aureus. These results indicated that the metabolic activity of intracellular S. aureus was not related to differences in gene expression within the macrophage population. However, we cannot rule out that heterogeneity in the capacity of individual phagosomes within the same cell or between cells to generate reactive oxygen species (ROS) (13) could affect the metabolic activity of the engulfed S. aureus, as these types of differences would not be revealed by an RNA-seq analysis.

**TABLE 2:** KEGG pathway annotation of upregulated DEGs in macrophages harboring either green or red S. aureus in comparison to uninfected macrophages

	Macrophages harboring green S. aureus		Macrophages harboring red S. aureus	
KEGG pathway	Adjusted *P* value	No. of genes	Adjusted *P* value	No. of genes
Cytokine-cytokine receptor interaction	1.90E−15	33	8.00E−15	30
TNF signaling pathway	2.00E−14	23	8.00E−15	22
Influenza A	1.90E−09	22	2.90E−08	19
Herpes simplex virus 1 infection	5.60E−08	22	2.90E−09	22
Toll-like receptor signaling pathway	5.90E−08	16	5.50E−08	15
Malaria	9.80E−07	11	2.80E−06	10
Chagas disease	3.90E−06	14	4.30E−06	13
Chemokine signaling pathway	1.10E−05	18	5.10E−06	17
Leishmaniasis	1.10E−05	11	2.10E−04	9
Measles	1.20E−05	15	1.20E−03	11
Tuberculosis	4.70E−05	16	n.s.^*a*^	n.s.
African trypanosomiasis	8.00E−05	8	n.s.	n.s.
Rheumatoid arthritis	8.10E−05	11	9.20E−04	9
NOD-like receptor signaling pathway	2.30E−04	9	6.30E−04	8
Inflammatory bowel disease	2.80E−04	9	n.s.	n.s.
NF-kappa B signaling pathway	2.90E−04	11	5.70E−04	10
Salmonella infection	2.90E−04	10	3.60E−03	8
Jak-STAT signaling pathway	3.90E−04	13	1.30E−04	13
Cytosolic DNA-sensing pathway	4.00E−04	9	1.20E−03	8
Transcriptional misregulation in cancer	1.20E−03	13	4.00E−04	13
Legionellosis	1.20E−03	8	n.s.	n.s.
Toxoplasmosis	2.30E−03	10	4.00E−03	9

a n.s., not significant.

10.1128/mbio.02316-22.4 TABLE S1 Genes exhibiting differential expression between macrophages harboring green S. aureus and uninfected macrophages. Download Table S1, XLSX file, 0.7 MB. Copyright © 2022 Lang et al. 2022 Lang et al. https://creativecommons.org/licenses/by/4.0/ This content is distributed under the terms of the Creative Commons Attribution 4.0 International license.

10.1128/mbio.02316-22.5 TABLE S2 Genes exhibiting differential expression between macrophages harboring red S. aureus and uninfected macrophages. Download Table S2, XLSX file, 0.7 MB. Copyright © 2022 Lang et al. 2022 Lang et al. https://creativecommons.org/licenses/by/4.0/ This content is distributed under the terms of the Creative Commons Attribution 4.0 International license.

### Transcriptional response of intracellular green or red S. aureus and S. aureus in the infection inoculum.

**FIG 4:**
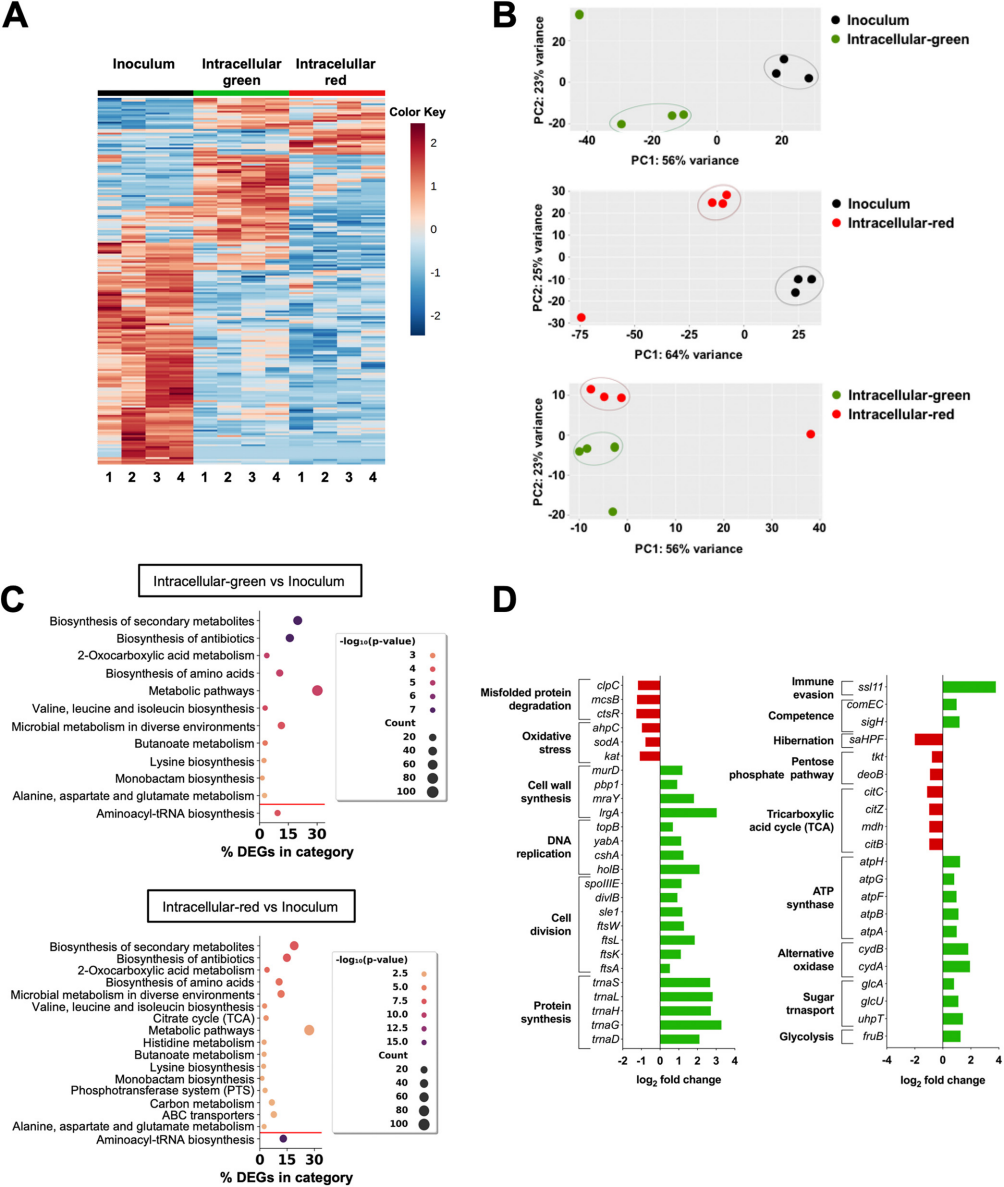
Transcriptional analysis of intracellular green or red S. aureus. (A) Heat map showing gene expression levels (top 500 genes) in intracellular green or red S. aureus as well as in S. aureus in the infection inoculum. The color key represents the *z* score normalized transcripts per million reads (TPM). (B) PCA clustering of the transcriptomes of intracellular green S. aureus versus S. aureus in the infection inoculum (upper panel), intracellular red S. aureus versus S. aureus in the infection inoculum (middle panel), and intracellular green versus intracellular red S. aureus (lower panel) based on a Euclidean distance matrix of normalized RNA-seq data. Circles enclose replicates that cluster together. Each dot represents one biological replicate. (C) Enriched KEGG pathways in genes with significantly greater expression (*P* < 0.05) and a log_2_-fold change of >1 (over the red line) or with significantly lower expression and a log_2_-fold change of <−1 (under the red line) in intracellular green S. aureus versus S. aureus in the infection inoculum (upper panel) or intracellular red S. aureus versus S. aureus in the infection inoculum (lower panel). The colors of the dots reflect the *P* values calculated by the DAVID software program, using a modification of Fisher’s exact test. The sizes of the dots reflects the number of genes in the pathway (count). (D) Expression levels of a set of DEGs between intracellular green and intracellular red S. aureus, shown as a log_2_-fold change. Positive values, depicted as green bars, indicate higher expression levels in green S. aureus, and negative values, depicted as red bars, indicate higher expression levels in red S. aureus.

The heat map depicted in Fig. 4A implied that the gene expression patterns differed between intracellular green bacteria, intracellular red bacteria, and bacteria in the infection inoculum. PCA clearly separated the transcriptional data sets of either green (Fig. 4B, upper panel) or red (Fig. 4B, middle panel) intracellular S. aureus from the transcriptome of S. aureus in the infection inoculum. This indicates that the gene expression profile of intracellular S. aureus differs significantly from that of the bacterial inoculum, independent of their metabolic activity. A PCA separated the gene expression data sets of intracellular green S. aureus and intracellular red S. aureus along the second principal component (PC2) due to the presence of an outlier sample in the red group that was responsible for the separation according to PC1 (Fig. 4B, lower panel).

A differential gene expression analysis comparing the transcriptional response between intracellular green or red S. aureus and the bacteria in the infection inoculum was then performed, and 563 DEGs were identified as significantly expressed (341 upregulated with a log_2_-fold change of >1 and 222 downregulated with a log_2_-fold change of <−1, with an adjusted *P* value of <0.05) for green S. aureus (Table S3), and 735 DEGs were identified as significantly expressed (378 upregulated and 357 downregulated) for red S. aureus (Table S4) in comparison to the inoculum bacteria. A core of 395 genes (266 upregulated and 129 downregulated) were differentially expressed by both groups of intracellular S. aureus compared to the bacteria in the infection inoculum (Table S5), indicating a common response of S. aureus to the intracellular milieu that is independent of metabolic state. A KEGG pathway enrichment analysis indicated that many of the core upregulated genes in intracellular S. aureus were related to the biosynthesis of secondary metabolites, the biosynthesis of several amino acids, and metabolic pathways (Fig. 4C; Tables S6 and S7). Among the genes related to amino acid biosynthesis were those encoding factors involved in the synthesis of methionine, lysine, and threonine (*lysC*, *asd*, *dapABD*) as well as those of the *ilv*-*leu* operon (*ilvA2BCD*, *leuBCD*), which encoded proteins involved in the biosynthesis of branched-chain amino acids (isoleucine, leucine, valine) (Fig. 4C; Tables S6 and S7). This indicates that the levels of these amino acids in the intracellular environment may not be sufficient to supplement the metabolic requirements of S. aureus and that the bacterium needs to synthesize them. Intracellular S. aureus can also obtain amino acids from host oligopeptides, and these can be imported via transporter systems, such as Opp-3 operon (*opp-3A-F*) (Table S5). The capacity to extract carbon sources from the host is critical for the intracellular survival of S. aureus, as shown by the upregulation of several transport systems for scavenging gluconate (*gntP*), sucrose (*scrA*), glucose (*glcB*), and maltose (*malF*) from the host (Table S5). The genes encoding the molecular chaperone DnaK and the co-chaperones DnaJ, GrpE, and GroEL/GroES, which play an important roles in protein folding and in the refolding of damaged proteins under stress conditions (14, 15), as well as those encoding members of the Clp ATPases, including ClpC, ClpB and their regulator CtsR, were also upregulated by both green and red intracellular S. aureus (Table S5). Since these factors are required for stress tolerance (16), the induction of these genes indicates that S. aureus faces high levels of stress in the intracellular compartment. A set of genes encoding important virulence factors, such as those encoding cytolysins, such as α-hemolysin (*hla*), ɣ-hemolysin subunits (*hlg*A, *hlg*B, *hlg*C), and two phenol-soluble modulins (PSM) beta (*psmβ2*), were also upregulated by both green and red intracellular S. aureus (Table S5). The roles of these toxins in the context of intercellular persistence and survival of S. aureus is rather controversial and highly depends on the host cell type. Thus, while some studies reported a role for α-hemolysin in S. aureus phagosomal escape and intracellular survival within macrophages (4) and cystic fibrosis lung CFT-1 cells (17), other studies have reported that α-hemolysin was not sufficient to lyse phagosomes in HeLa (18) or in upper airway epithelial cells (19). The expression of the genes encoding components of the accessory gene regulator (Agr), the most prominent regulatory system of S. aureus (20), were also upregulated by both green and red intracellular S. aureus (Table S5). The expression of Agr has been shown to be crucial for the intracellular survival of S. aureus within macrophages (4).

10.1128/mbio.02316-22.6 TABLE S3 Genes exhibiting differential expression between intracellular green S. aureus and S. aureus in the infection inoculum. Download Table S3, XLSX file, 0.1 MB. Copyright © 2022 Lang et al. 2022 Lang et al. https://creativecommons.org/licenses/by/4.0/ This content is distributed under the terms of the Creative Commons Attribution 4.0 International license.

10.1128/mbio.02316-22.7 TABLE S4 Genes exhibiting differential expression between intracellular red S. aureus and S. aureus in the infection inoculum. Download Table S4, XLSX file, 0.1 MB. Copyright © 2022 Lang et al. 2022 Lang et al. https://creativecommons.org/licenses/by/4.0/ This content is distributed under the terms of the Creative Commons Attribution 4.0 International license.

10.1128/mbio.02316-22.8 TABLE S5 Genes exhibiting differential expression in both green and red intracellular S. aureus respect to S. aureus in the infection inoculum. Download Table S5, XLSX file, 0.1 MB. Copyright © 2022 Lang et al. 2022 Lang et al. https://creativecommons.org/licenses/by/4.0/ This content is distributed under the terms of the Creative Commons Attribution 4.0 International license.

10.1128/mbio.02316-22.9 TABLE S6 KEGG pathways enriched in genes with significantly greater expression in intracellular green S. aureus versus S. aureus in the infection inoculum. Download Table S6, XLSX file, 0.01 MB. Copyright © 2022 Lang et al. 2022 Lang et al. https://creativecommons.org/licenses/by/4.0/ This content is distributed under the terms of the Creative Commons Attribution 4.0 International license.

10.1128/mbio.02316-22.10 TABLE S7 KEGG pathways enriched in genes with significantly greater expression in intracellular red S. aureus versus S. aureus in the infection inoculum. Download Table S7, XLSX file, 0.02 MB. Copyright © 2022 Lang et al. 2022 Lang et al. https://creativecommons.org/licenses/by/4.0/ This content is distributed under the terms of the Creative Commons Attribution 4.0 International license.

A KEGG pathway enrichment analysis of downregulated genes indicated that the aminoacyl-tRNA biosynthesis pathway was downregulated in both intracellular green and red S. aureus, relative to the bacteria in the inoculum (Fig. 4C; Tables S6 and S7). Since the expression of aminoacyl-tRNA synthesis is regulated by the T-box control system (21), the downregulation of this pathway in both green and red S. aureus may indicate an absence of uncharged tRNAs, most likely due to a sufficient supply of amino acids.

Next, we analyzed the DEGs between intracellular green and red S. aureus. A total of 171 genes were found to be differentially expressed between the green and red bacterial subpopulations, of which 124 exhibited higher expression in green S. aureus than in red S. aureus, whereas 47 exhibited higher expression in red S. aureus than in green S. aureus (Table S8). Among the genes exhibiting higher expression in green S. aureus were those involved in cell division (*ftsL*, *ftsW*, *ftsK*, *ftsA*, *sle1*, *divlB*, *spoIIIE*, *mraZ*), DNA replication (*holB*, *cshA*, *yabA*, *topB*), and cell wall synthesis (*lrgA*, *mraY*, *pbp1*, *murD*) (Fig. 4D; Table S8), which corroborated that the green S. aureus were undergoing cell division much more actively than were the red S. aureus. Several genes encoding tRNAs were also expressed to a greater extent by the green S. aureus (Fig. 4D; Table S8), which may indicate a greater consumption of amino acids by this group.

10.1128/mbio.02316-22.11 TABLE S8 Genes exhibiting differential expression between intracellular green and red S. aureus. Download Table S8, XLSX file, 0.04 MB. Copyright © 2022 Lang et al. 2022 Lang et al. https://creativecommons.org/licenses/by/4.0/ This content is distributed under the terms of the Creative Commons Attribution 4.0 International license.

**FIG 5:**
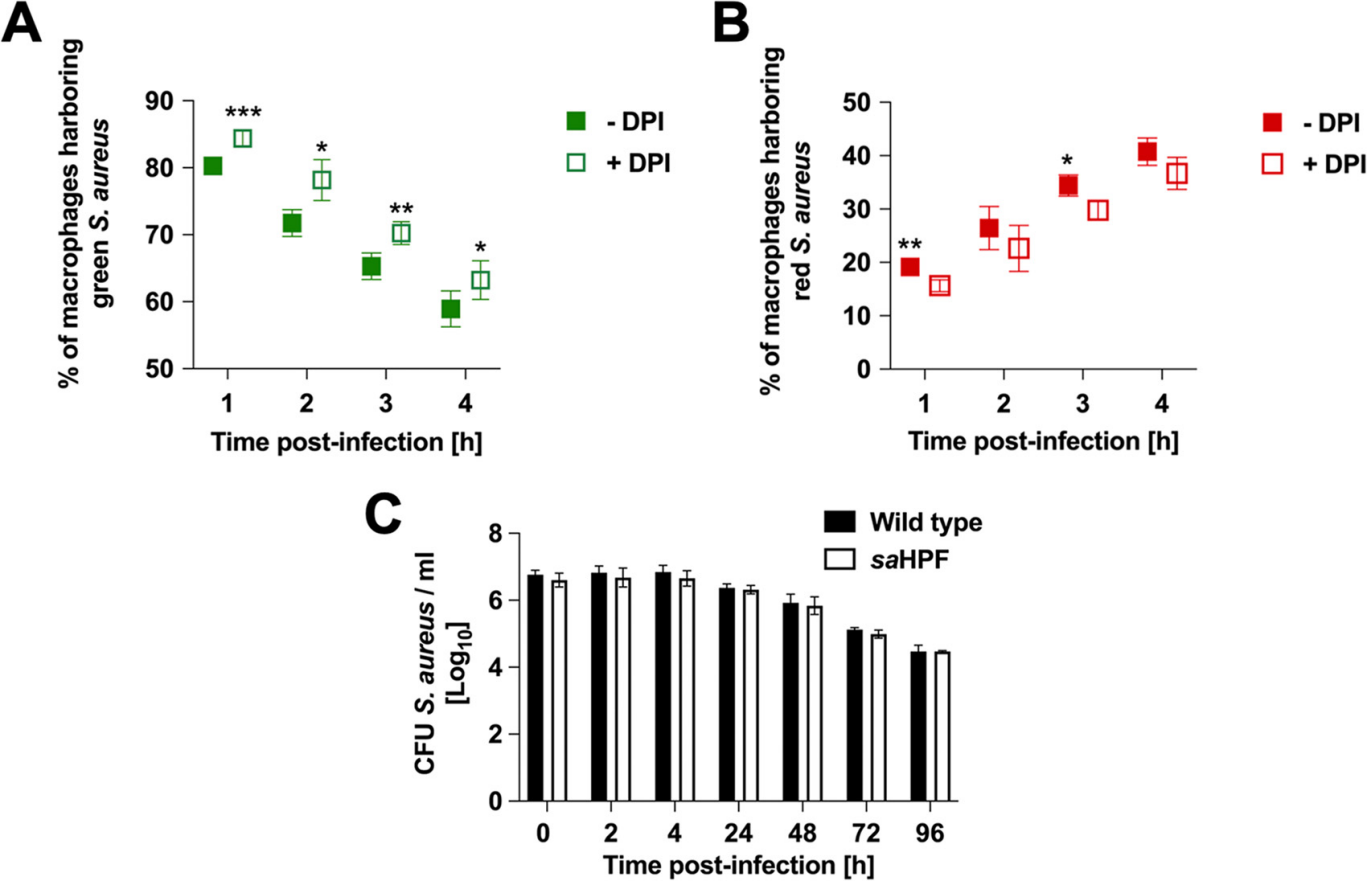
Effect of DPI treatment on the metabolic activity of intracellular S. aureus. (A) Percentage of macrophages harboring green S. aureus in the presence (open symbols) or absence (closed symbols) of DPI. (B) Percentage of macrophages harboring red S. aureus in the presence (open symbols) or absence (closed symbols) of DPI. (C) Viability of intracellular S. aureus wild-type and saHPF mutant strains during the course of infection. Each bar represents the average ± standard deviation of three independent experiments. *, *P <* 0.05; **, *P <* 0.01; ***, *P <* 0.001.

Among the genes with higher expression in red S. aureus were those involved in stress responses, such as oxidative stress (*kat*, *sodA*, *ahpC*) and misfolded protein degradation (*ctsR*, *mcsB and clpC*) (Fig. 4D; Table S8). Because these genes are induced in response to environmental stresses (22), it can be anticipated that red S. aureus encountered harsher conditions in the intracellular niche, such as higher levels of ROS, than did the green S. aureus and therefore needed to induce a more extensive oxidative stress response to neutralize them. To test this hypothesis, we compared the percentage of macrophages harboring green or red S. aureus in the presence or absence of the NADPH oxidase inhibitor diphenyleneiodonium chloride (DPI). ROS generated by the NADPH oxidase is one of the microbicidal mechanisms used by macrophages to kill phagocytosed bacteria (23). The inhibition of NADPH oxidase after treatment with DPI resulted in a significant increase in the percentage of macrophages harboring green bacteria (Fig. 5A) and in a concomitant decrease in the percentage of macrophages harboring red bacteria (Fig. 5B). However ROS production through the activation of the NADPH oxidase does not seem to be the only factor driving the emergence of red S. aureus, as the number of macrophages containing green bacteria continued to decline and the number of macrophages containing red bacteria continued to increase over time, irrespective of DPI treatment.

The differential expression of genes related to metabolism indicated that the nutrient availability differed between the intracellular environments in which the green and red S. aureus were located. Thus, the green S. aureus exhibited higher expression of genes encoding members of the alternative oxidase (*cydAB* and *qoxA*), suggesting that they use aerobic respiration to a larger extent than do the red S. aureus (Fig. 4D; Table S8). In addition, genes encoding enzymes involved in glycolysis (*fruB*) and sugar transport (*uhpT*, *glcU*, *glcA*) exhibited greater expression in green S. aureus, whereas genes related to the tricarboxylic acid (TCA) cycle (*mdh*, *citBCZ*) and the pentose phosphate pathway (*deoB*, *tkt*) were expressed to a greater extent in red S. aureus (Fig. 4D; Table S9). This suggests that red S. aureus may be located in a nutrient-poor intracellular compartment in which TCAs cycle activity is required for the catabolism of nonpreferred carbon sources (24). Notably, the gene encoding the alternative sigma factor H (*sigH*) was expressed to a higher extent by green S. aureus than by red S. aureus (Fig. 4D; Table S8). The detection of *sigH* expression in intracellular S. aureus is of interest, as its expression is not detectable under standard laboratory culture conditions (25) and little is known about its function (26). SigH is required for the induction of competence genes in S. aureus (26), and bacteria expressing SigH become competent for transformation by foreign DNA (25). SigH induces the expression of the *com* operon, which encodes proteins required for natural genetic competence (26). In this regard, the genetic competence genes *comEC* exhibited greater expression in green S. aureus than in red S. aureus (Fig. 3C; Table S8). The gene encoding SSL11 was found to be expressed almost exclusively by green S. aureus (Fig. 4D; Table S8). SSL11 has been shown to influence neutrophil functions, including neutrophil activation (27), but so far, no function for SSL11 in S. aureus intracellular survival has been reported.

We also observed that the gene *saHPF* encoding the staphylococcal ribosome hibernation promoting factor (saHPF) exhibited significantly greater expression in red S. aureus than in green S. aureus (Fig. 4D; Table S8). During extreme stress conditions and nutrient deprivation, the 70S ribosomes are converted into inactive 100S ribosome dimers by association with saHPF, which helps the pathogen to reduce energy by silencing protein translation (28). Upon introduction into a favorable environment, 100S ribosomes readily dissociate and begin protein synthesis (29). Furthermore, saHPF was the only protein exhibiting increased expression levels in S. aureus HG001 after internalization within human bronchial epithelial cells (30). Since it has been reported that a mutant strain of S. aureus deficient in the expression of *saHPF* had extremely low survival during long-term culture in nutrient limited conditions (28), we determined the relevance of saHPF for the capacity of S. aureus to persist within macrophages. For this purpose, a mutant strain of S. aureus SH1000 deficient in the expression of saHPF (*sa*HPF) was generated and used for infecting macrophages. As shown in Fig. 5C, a lack of *saHPF* expression did not affect the capacity of S. aureus to survive and persist within macrophages, as the numbers of *sa*HPF bacteria recovered from macrophages at different times postinfection were comparable to that of the wild-type strain. This indicates that saHPF is not strictly required for S. aureus intracellular persistence in our infection model and experimental time frame.

In summary, we envision a scenario in which a proportion of S. aureus taken up by macrophages localizes within phagolysosomes that undergo progressive maturation, involving fusion with acidic lysosomal compartments and production of high levels of ROS (31). These bacteria will survive within these compartments by inducing a rapid stress response to counteract the harsh environment and by finally entering into a dormant-like state by reducing energy-consuming processes. Another fraction of phagocytosed S. aureus will actively proliferate and will eventually disrupt the macrophage to get released and establish a new focus of infection. Thus, to overcome frequent therapeutic failure and infection recurrence, innovative treatment strategies need not only to target actively replicating bacteria but also to have the capacity to penetrate into the intracellular compartment and target dormant-like persistent S. aureus.

## MATERIALS AND METHODS

### Bacterial strains.

The S. aureus strains used in this study were S. aureus SH1000 wild type, S. aureus SH1000 pKikume (10), and the HPF-deficient isogenic S. aureus mutant strain (*sa*HPF) generated in this study. Staphylococci were grown in brain heart infusion medium (BHI) overnight with steady shaking (150 rpm) at 37°C. Bacterial cultures were refreshed at a ratio of 1:100 in BHI medium, grown to mid-log-phase, collected by centrifugation for 10 min at 16,000 × *g*, and adjusted to the desired concentration.

Photoconversion of S. aureus pKikume from green to red fluorescence was performed by a 90 sec pulse of violet light (UV) at a 405 nm wavelength using a 10 × 10 LED array (half-viewing angle: 15°; radiant power: 10 mW).

### Generation of HPF-deficient S. aureus mutant strain (*sa*HPF).

The transposon insertion mutant in the *saHPF* gene (locus ID SAOUHSC_00767 in S. aureus NCTC8325) was identified in the Nebraska Transposon Mutant Library (32) with strain identification NE838 (locus ID SAUSA300_RS03960 in S. aureus USA300_FPR3757, gene symbol yfiA). The transposon was transduced into S. aureus SH1000 using phage Φ11, and the insertion was confirmed by PCR.

### Culture of immortalized macrophages.

Immortalized bone marrow macrophages from C57BL/6 mice (33) were kindly provided by M. Brinkmann, HZI Braunschweig, Germany. Macrophages were cultured in DMEM/Gibco supplemented with 10% FCS at 37°C and 5% CO_2_ in cell culture dishes.

### Cell death assay.

Cell death was determined by measuring the levels of lactate dehydrogenase (LDH) by using a CytoTox 96 Non-Radioactive Cytotoxicity Kit according to the manufacturer’s recommendations (Promega).

### Infection assay.

Macrophages were infected with S. aureus at a multiplicity of infection (MOI) of 5:1 and were incubated for 2 h to allow the macrophages to internalize the bacteria. The medium was then replaced by medium containing 100 μg/mL gentamicin (Gibco) and 5 μg/mL lysostaphin (Sigma-Aldrich) to kill noningested extracellular bacteria. This was considered the 0 h time point in all experiments. Infected macrophages were further incubated in the presence of antibiotics.

For the determination of bacterial intracellular viability, macrophages were infected as described and lysed at progressing times postinfection by adding _dd_H_2_O supplemented with 0.1% Triton X-100 for 5 min at RT. Serial dilutions of cell lysates were plated on blood agar, and bacterial colonies were counted after overnight incubation at 37°C.

The photoconversion of intracellular S. aureus pKikume was performed by subjecting the infected macrophages to a 90 sec pulse of violet light (UV) at a 405 nm wavelength using a 10 × 10 LED array (half-viewing angle: 15°; radiant power: 10 mW).

For analyzing the phagocytic capacity of macrophages after UV exposure, the macrophages received a 90 sec pulse of UV at a 405 nm wavelength and were infected with S. aureus at a 5:1 MOI 1 h after treatment. After 2 h postinfection, the macrophages were lysed, and serial dilutions of cell lysates were plated on blood agar. Bacterial colonies were counted after overnight incubation at 37°C.

In some experiments, the macrophages were treated with the NADPH oxidase inhibitor diphenyleneiodonium (DPI, Sigma-Aldrich) at 10 μM for 1 h prior to infection.

### Flow cytometry and cell sorting.

For the flow cytometry analysis, macrophages infected with S. aureus pKikume were subjected to a 90 sec pulse of UV at a 405 nm wavelength 30 min before sampling. The macrophages were then collected and analyzed by flow cytometry in a LSR II SORP flow cytometer (BD Biosciences). The red mKikume fluorescence signal was measured at 561 nm excitation and 582/15 nm emission, and the green mKikume fluorescence signal was measured at 488 nm excitation and 525/50 nm emission. The data were analyzed by using the FlowJo X software (FlowJo, LLC).

The sorting of macrophages subpopulations harboring either red or green S. aureus was performed using a FACSAria-II SORP cell sorter (BD Biosciences).

### ATP measurements.

Green or red S. aureus bacteria were released from macrophages after sorting by treating them with 0.1% (wt/vol) TritonX-100, and the ATP was determined in both bacterial populations by using an ATP determination kit (Thermo Fisher Scientific). Briefly, bacteria were collected by centrifugation, washed in 50 mM Tris-HCl, and resuspended in 50 μL phosphate/EDTA buffer. After snap-freezing the samples in liquid nitrogen, the suspension was thawed to RT, and the bacteria were lysed using Bacterial Lysis Mix. A 50 μL volume of each lysate was incubated with 50 μL of luciferin/luciferase working solution, and bioluminescence was measured using a SpectraMax M3 548 Microplate Reader (Molecular Devices). Control wells containing culture medium alone were used for background determination. The amount of ATP measured was expressed in relative light units (RLU) per viable bacterium after normalization by the colony forming units (CFU) determined by plating.

### Confocal laser scanning microscopy and Widefield time lapse imaging.

Macrophages were seeded in a μ-Slide 8-Well chamber (Ibidi) and preloaded with Alexa Fluor 647-labeled dextran (Invitrogen) to label lysosomal compartments. The following day, the cells were thoroughly washed and infected with S. aureus pKikume as described above. The photoconversion of intracellular S. aureus pKikume was performed 30 min before microscopy by a 90 sec pulse of violet light (UV) at a 405 nm wavelength using a 10 × 10 LED array (Strato, half-viewing angle: 15°; radiant power: 10 mW), and the macrophages were examined using an inverted confocal laser scanning microscope SP5 (Leica Microsystems).

Widefield time lapse imaging of S. aureus pKikume within macrophages was performed using an Olympus BX61 microscope equipped with a 20× dry objective and an F-view camera that was controlled by the cellR software (Version 2.0, Olympus Biosystems). A mercury arc lamp served as the illumination source and was combined with excitation filters at 480/17 nm and 556/20 nm, a FITC/Cy3/Cy5 triple-band dichroic beamsplitter, and emission filters at 520/28 nm and 617/73 nm in order to determine the fluorescence of mKikume green and red, respectively. The photoconversion of the intracellular bacteria was performed using an illumination of 15 mW/cm^2^ for 1 min using the 436/10 nm excitation filter of the microscope’s epifluorescence illumination.

### RNA extraction and purification.

Total RNA was isolated from either S. aureus*-*infected macrophages, S. aureus grown in BHI to mid-log-phase (inoculum), or uninfected macrophages using the PureLink RNA minikit (Invitrogen) according to the manufacturer’s instructions. Mechanical sample disruption was performed prior to RNA extraction using lysing matrix B tubes (MP Biomedicals) and a FastPrep-24 instrument at an intensity of 6.5 m/s for 3 × 30 sec with 1 min resting on ice between the runs. Sample lysates were then subjected to PureLink on-column DNase treatment (Invitrogen) to remove DNA contamination according to the manufacturer’s instructions. The quality and quantity of the RNA samples were determined using a NanoDrop One spectrophotometer and an Agilent 2100 Bioanalyzer. DNA-depleted RNA samples were further subjected to rRNA depletion using the Ribo-Off rRNA depletion kit for bacteria and human/mouse/rat (Vazyme). The depletion of both eukaryotic and bacterial rRNA was performed simultaneously, following the manufacturer’s instructions recommended for bacteria and with an additional 1 μL rRNA probe for human/mouse/rat added to the hybridization mix.

### RNA-seq library preparation and sequencing.

The cDNA libraries were prepared from mRNA of each sample using the NEBNext Ultra II Directional RNA Library Prep Kit followed by sequencing on an Illumina NovaSeq 6000 (Illumina). The parameters for sequencing were set to generate 100 million reads per sample of 150 bp in length and paired ended, as well as 50 million reads per sample for S. aureus in pure culture.

### RNA-seq data processing.

The RNA-seq data sets were quality controlled using FastQC followed by the *in silico* removal of rRNA sequencing transcripts (reads) that remained despite rRNA depletion by Ribo-Off and were subjected to quality trimming, adapter removal, and length filtering using Trimmomatic. Reads were trimmed from the 3′-end if the Phred quality score for a base call was ≤30 (Phred + 33) and entirely excluded if the read length was ≤50. The sequencing reads were mapped to the reference genome of Mus musculus assembly GRCm38.p6 (GCA_000001635.26) as well as to the reference genome of S. aureus strain NCTC 8325 using the Spliced Transcripts Alignment to Reference (STAR) software. The minimal mapping length was set to 20, and the number of allowed mismatches was set to 6. The mapped reads aligned to the murine and the staphylococcal reference genome were outputted in binary BAM format sorted by coordinates. Subsequently, the number of transcripts aligned to a certain gene were counted using feature counts. For the downstream analysis, only the uniquely mapped reads were kept, as multiple counting can arise not only from a biological reason but also from technical artifacts.

Raw sequence read files can be found at the European Nucleotide Archives (ENA) under the project ID ERX6202369.

### Statistical analysis of transcriptional data.

The identification of DEGs for the murine and bacterial transcriptome was performed using the DESeq2 package in R. DEGs with an adjusted *P* value below 0.05 were considered statistically significant. The transcriptional profiles of each sample were compared based on a Euclidean distance matrix using a principal-component analysis. Gene lists of all significantly expressed genes between the different conditions were used as an input for a KEGG pathway analysis using DAVID. An analysis of variance (ANOVA) test with Tukey’s *post hoc* test or a *t* test was used for comparisons between groups. *P* values of <0.05 were considered indicative of a statistically significant result.
