# Supplementary material for: A Photoconvertible Reporter System for Bacterial Metabolic Activity Reveals That Staphylococcus aureus Enters a Dormant-Like State to Persist within Macrophages
Source: mBio. 2022 Sep 14;13(5):e02316-22. doi: 10.1128/mbio.02316-22 (PMC9600638; doi:10.1128/mbio.02316-22)
Supplement: FIG S1 [file mbio.02316-22-s0001.pdf]

Supplementary Figure S1

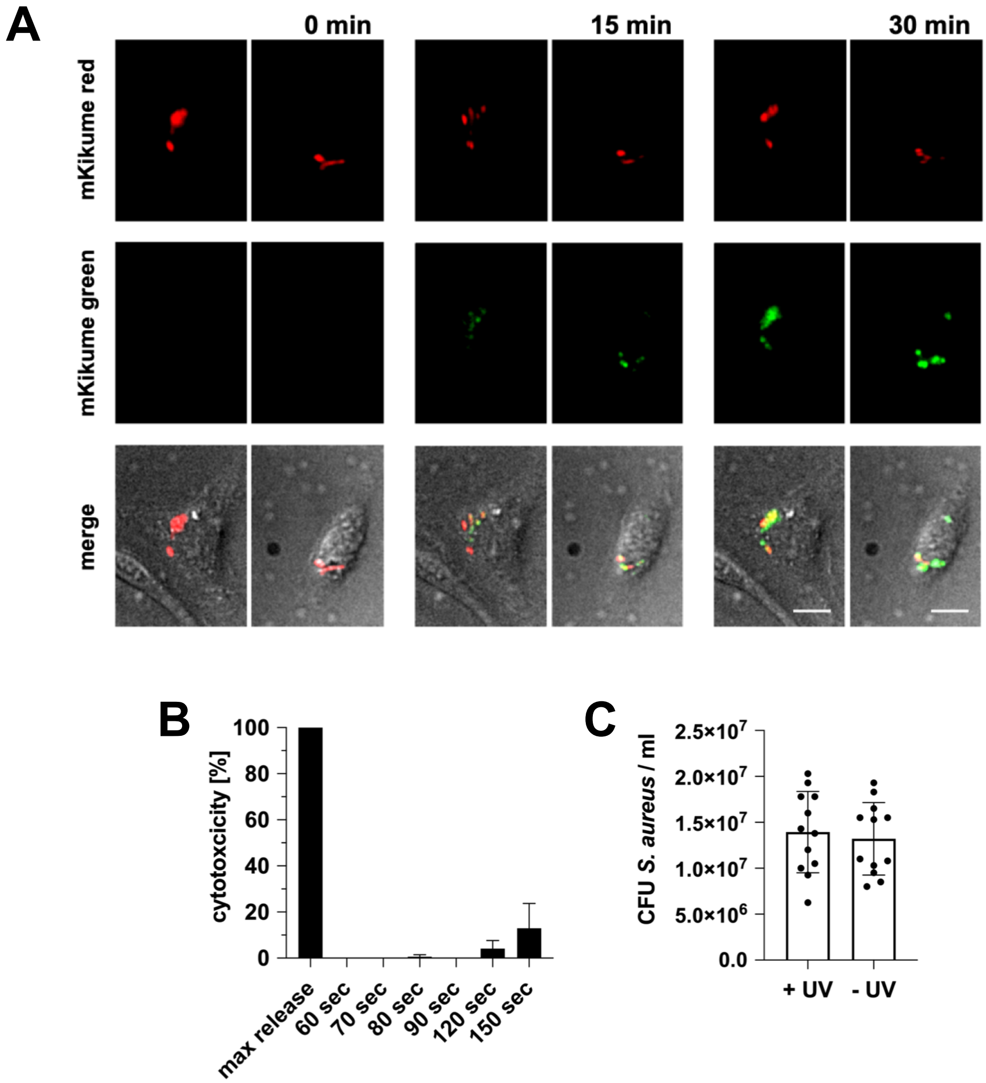

Figure S1. (A) Widefield time lapse imaging of *S. aureus* pKikume within macrophages after photoconversion. Time-lapse widefield microscopy of macrophage infection was performed using a Olympus BX61 microscope equipped with a 20x dry objectives, controlled by the cellR software (Version 2.0, Olympus Biosystems). A mercury arc lamp served as illumination source and was combined with excitation filters at 480/17 nm and 556/20 nm, a FITC/Cy3/Cy5 triple-band dichroic beamsplitter and emission filters at 520/28 nm and 617/73 nm. Scale bar, 10 µm. (B) Lactate dehydrogenase (LDH) released in the culture supernatant of *S. aureus*-infected macrophages after exposure to UV light for increasing periods of time. Results are displayed as percentage of the maximum LDH release achieved after disruption of macrophages with 1% Triton X-100. (C) Quantification of viable *S. aureus* bacteria within untreated or UV light-treated macrophages after 2 h phagocytosis. For UV light treatment, macrophages were exposed to UV light for 90 s and infected with *S. aureus* 1 h thereafter. Each bar represents the average ± SD of three independent experiments.
